# Inconsistent reversal of HIV-1 latency ex vivo by antigens of HIV-1, CMV, and other infectious agents

**DOI:** 10.1186/s12977-020-00545-x

**Published:** 2020-11-23

**Authors:** Thomas Vollbrecht, Aaron O. Angerstein, Bryson Menke, Nikesh M. Kumar, Michelli Faria de Oliveira, Douglas D. Richman, John C. Guatelli

**Affiliations:** 1 Department of Medicine, University of California San Diego, La Jolla, CA USA; 2 VA San Diego Healthcare System, San Diego, CA USA; 3 Department of Biological Sciences, University of California San Diego, La Jolla, CA USA; 4 Department of Pathology, University of California San Diego, La Jolla, CA USA

**Keywords:** Latency, HIV-1, Antigen, CD4 T cell

## Abstract

**Background:**

A reservoir of replication-competent but latent virus is the main obstacle to a cure for HIV-1 infection. Much of this reservoir resides in memory CD4 T cells. We hypothesized that these cells can be reactivated with antigens from HIV-1 and other common pathogens to reverse latency.

**Results:**

We obtained mononuclear cells from the peripheral blood of antiretroviral-treated patients with suppressed viremia. We tested pools of peptides and proteins derived from HIV-1 and from other pathogens including CMV for their ability to reverse latency ex vivo by activation of memory responses. We assessed activation of the CD4 T cells by measuring the up-regulation of cell-surface CD69. We assessed HIV-1 expression using two assays: a real-time PCR assay for virion-associated viral RNA and a droplet digital PCR assay for cell-associated, multiply spliced viral mRNA. Reversal of latency occurred in a minority of cells from some participants, but no single antigen induced HIV-1 expression *ex vivo* consistently. When reversal of latency was induced by a specific peptide pool or protein, the extent was proportionally greater than that of T cell activation.

**Conclusions:**

In this group of patients in whom antiretroviral therapy was started during chronic infection, the latent reservoir does not appear to consistently reside in CD4 T cells of a predominant antigen-specificity. Peptide-antigens reversed HIV-1 latency ex vivo with modest and variable activity. When latency was reversed by specific peptides or proteins, it was proportionally greater than the extent of T cell activation, suggesting partial enrichment of the latent reservoir in cells of specific antigen-reactivity.

## Background

By preventing cells from becoming newly infected, combined antiretroviral therapy (cART) can suppress the replication of the human immunodeficiency virus-1 (HIV-1) to nearly undetectable levels. However, latently infected cells persist, are remarkably stable, and once activated yield new infectious virus [1–3]. Consequently, interruption of cART is followed by a relapse of viral replication and viremia, which ultimately causes depletion of CD4 T cells and immunodeficiency if left untreated [4].

Latently infected cells form a “viral reservoir” that is established early after infection with replication-competent proviruses integrated into the genomes of CD4 cells, with a substantial proportion in resting memory CD4 T cells [5–8]. During acute infection, HIV-1 causes immune activation, preferentially infecting activated HIV-1 specific CD4 T cells [9]. This suggests the possibility that the viral reservoir is biased toward such HIV-specific memory cells.

A currently favored approach to eradicate latently infected T cells is to reactivate viral transcription using small molecules as latency reverting agents (LRAs) while maintaining cART [10]. The goal of this approach is to activate latent viruses that are integrated into host memory CD4 T cells so that the cells die from viral cytopathic effect or are eliminated by immune surveillance. These strategies have so far shown very limited success [11–14]. Agents under investigation as LRAs include various types of protein kinase C (PKC) activators (for example, bryostatin-1), HDAC inhibitors (for example, panobinostat and romidepsin), and BRD4 inhibitors (for example, JQ1), among others [15–19]. Most of these LRAs lack specificity for the cells that contain the viral reservoir. Consequently, some of them have a potential for toxicity; for example, agents targeting PKC-signaling might broadly affect cellular metabolism [20, 21]. In contrast, activation of latently infected memory CD4 T cells by their cognate antigens is specific. However, the effectiveness of an antigen as a LRA will likely depend on the fraction of the latent reservoir that resides in cells that respond to it.

Since HIV-1 preferentially infects activated CD4 T cells [5, 9], and since the reservoir is partly established early during infection when most of those cells are responding to HIV, many reservoir cells might be HIV-specific. Alternatively, if HIV-infection is left untreated while CD4 T cells are responding to either chronic persistent infections such as those caused by CMV or EBV or recurrent infections such as influenza, then reservoir cells might be specific for those pathogens. More generally, if reservoir cells have a limited breadth of antigen specificity, then only a few antigens might be needed to reverse latency in the majority of cells while activating only a small fraction of the CD4 T cell population.

In this study, we hypothesized that the latent reservoir can be reactivated using antigens to activate specific memory CD4 T cells harboring latent provirus. We evaluated whether peptide antigens specific to HIV, as well as to common pathogens such as CMV, could act as LRAs to reactivate the HIV-1 reservoir without causing general T cell activation. Support for this hypothesis came from previous studies suggesting that peptide antigens of HIV-1 induced viral protein expression in CD4 T cells from participants with suppressed viremia [22–24]. Additionally, we tested the hypothesis that the presentation of antigens by dendritic cells optimizes the reversal of latency (reviewed in [25]). We found that in some cases antigenic peptides or proteins reversed latency, but they rarely did so to a substantial extent, and no particular antigen was active in all cases. When peptides or protein antigens reversed latency, they did so to an extent greater than would be predicted based on their induction of T cell-activation. This suggests that in occasional individuals the latent reservoir is disproportionally present in cells of specific antigen-reactivity, a finding potentially consistent with clonal expansion of specific memory CD4 T cells.

## Results

### Study participants and cell preparation

We obtained up to 300 ml of peripheral blood from 19 HIV-1 positive participants (one on two occasions); three participants were ultimately excluded from the data presented, because their cells did not show evidence of latency reversal after stimulation with antibodies to CD3 and CD28 (the positive T cell activation control). All participants had a CD4 T cell count of at least 500 cells/µl of peripheral blood and fully suppressed viremia (less than 20 copies/ml) for at least one year. For the participants for whom data are available, HIV-1 infection was diagnosed 5 to over 27 years before their blood was obtained for this study (Table 1).

**Table 1:** Clinical data for the study participants

PID	Age	Gender	Race	Date of infection	Date of diagnosis	ART start	CMV+	EBV+	Influenza vaccine
1	52	Male	White, non-hispanic	11/19/1989	6/1/1992	8/1/1998	n.a.	n.a.	n.a.
2	58	Male	White, non-hispanic	n.a.	1993	1 mo post dx	n.a.	n.a.	n.a.
3	62	Male	Black	n.a.	1996	n.a.	n.a.	n.a.	n.a.
4	59	Male	American indian	1996	2006	2006	n.a.	n.a.	n.a.
5	48	Female	Hispanic	n.a.	2006	3/1/2007	n.a.	n.a.	n.a.
6	63	Female	White, non-hispanic	2000	2000	1 mo post dx	n.a.	n.a.	n.a.
7	66	Female	White, non-hispanic	2007	2010	1 mo post dx	n.a.	n.a.	n.a.
8	53	Male	Hispanic	7/2013	7/2013	7/2013	n.a.	n.a.	n.a.
9	58	Male	White, non-hispanic	n.a.	9/11/2007	10/30/2007	n.a.	n.a.	n.a.
10	50	Male	White, non-hispanic	n.a.	1/15/1997	12/12/2001	n.a.	n.a.	n.a.
11	50	Male	White, non-hispanic	2/2007	2/2008	2/2018	n.a.	n.a.	n.a.
12	51	Male	White, non-hispanic	11/2001	2001	n.a.	n.a.	n.a.	n.a.
13	59	Male	Hispanic	1992	1998	2002	n.a.	n.a.	n.a.
14	56	Male	Hispanic	2004–06	2009	2009	n.a.	n.a.	n.a.
15	73	Male	White, non-hispanic	1994	5/7/2003	5/2003	n.a.	n.a.	n.a.
16	55	Male	Black	n.a.	n.a	10/2006	n.a.	n.a.	n.a.
17	40	Male	n.a.	n.a.	6/2014	7/2014	n.a.	n.a.	11/2014 ; 10/2015 ; 10/2016 ; 9/2017
18	62	Male	n.a.	n.a.	1991	1991	Positive	n.a.	2000–2012; 11/2013 11/2014; 10/2016
19	59	Male	White	n.a.	1991	1991	Negative (2008)	n.a.	2009–2014; 11/2015 10/2017

PID, Patient ID; n.a., data not available; dx, diagnosis

We isolated peripheral blood mononuclear cells (PBMC) by density centrifugation. Constitutive expression of MHC class-II molecules is confined to professional antigen presenting cells (APCs). To ensure optimal antigen presentation to CD4 T cells in the context of MHC class-II, we kept professional APCs—B cells and monocytes—in the cell population by depleting CD8 T cells from the PBMC rather than isolating the CD4 T cells. Our rationale for removing CD8 T cells was to avoid potential killing of reactivated reservoir cells as well as the potential secretion of suppressive mediators. CD8 T cell depletion from participant PBMC was confirmed by flow cytometry, and residual CD8 T cells were usually < 0.1% (data not shown).

### Identification of antigen specific CD4 T cell responses by IFN-γ ELISpot assay

Production of interferon (IFN)-γ by CD4 T cells is a hallmark of the Th1-type CD4 T cell phenotype and is typically associated with an effective host defense against intracellular pathogens (reviewed in [26]). Therefore, we first used an IFN-γ enzyme-linked immune absorbent spot (ELISpot) assay to evaluate the ability of peptide pools to activate and induce an immune response in the CD4 T cells within the CD8 depleted cell populations.

**Fig. 1:**
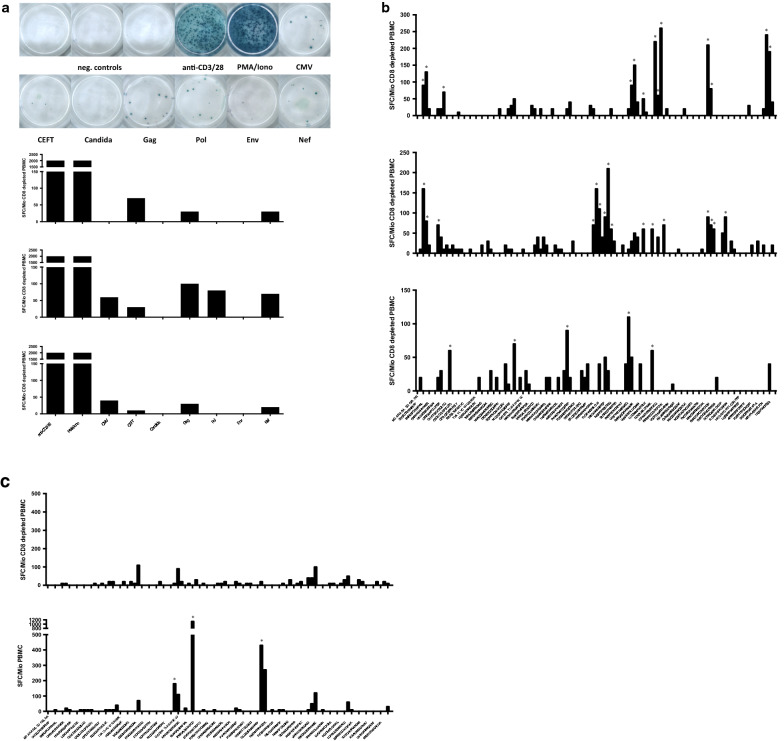
**IFN-γ ELISpot**. **a** CD4 T cell response to pooled 15mer peptides with 11 amino acid overlap, specific for: CMV pp65 (138 peptides), CEFT (14 peptides selected from defined HLA class-II restricted T-cell epitopes of *Clostridium tetani*, Epstein-Barr virus (HHV-4), human cytomegalovirus (CMV; HHV-5), and Influenza A), HIV-1 consensus B Gag (123 peptides), HIV-1 consensus B Env (211 peptides), HIV-1 consensus B Pol (249 peptides), and HIV-1 consensus B Nef (49 peptides). Example wells are shown for each peptide pool; the examples are from different participants. Below are the bar graphs of three study participants depicting the IFN-γ response to the tested peptide pools. **b** CD4 T cell responses of three participants towards 123 individual overlapping 15mer HIV-1 consensus B Gag peptides. The x-axis shows the sequence of every other single peptide. *SFC* spot forming cells. Marked by asterisks are the respective peptides that were subsequently used for the participants’ customized ELISpot-selected Gag peptide pools. **c** Comparison of PBMC (bottom) and CD8 depleted PBMC from the same blood donor. Marked by asterisks are peptides that were associated with CD8 T cell responses that were not present in the assay using CD8 depleted PBMC

We screened IFN-γ production in response to peptide pools containing 15mer overlapping peptides from: (1) Cytomegalovirus (CMV) matrix protein 65 (pp65); (2) *Candida albicans* mannoprotein MP65; (3) a mixture of 14 previously identified optimal MHC class-II restricted epitopes from human cytomegalovirus (HHV-5), Epstein-Barr virus (HHV-4), influenza A, and *Clostridium tetani* toxoid (pool named CEFT); (4) HIV-1 Gag; (5) HIV-1 Pol; (6) HIV-1 Env; and (7) HIV-1 Nef. For each condition, we stimulated 100,000 CD8 depleted PBMC with 1 µg/ml per individual peptide. We used anti-CD3/CD28-coated beads as well as phorbol myristate acetate in combination with ionomycin (PMA/Iono) as a positive control for maximal T cell stimulation. Both positive controls typically saturated the signals on the ELISpot plate and did not allow quantification (Fig. 1a). We used DMSO as a negative control, because the peptide pools and individual peptides were reconstituted in DMSO. Wells treated with DMSO typically showed < 10 spot forming cells (SFC)/million cells. IFN-γ responses of varying magnitudes were measured for these peptide pools in CD8 depleted PBMCs from a subset of participants (Fig. 1a). For three participants, we tested the 123 Gag 15mer peptides singly at a concentration of 12.5 µg/ml per peptide with 100,000 CD8 depleted PBMC per well to identify the specific peptides within the pool that induced IFN-γ production in the CD4 T cells (Fig. 1b). Individual peptides that yielded > 50 SFC/million CD8 depleted PBMC were subsequently pooled to generate a participant-customized ELISpot selected Gag peptide pool used in some of the latency reactivation experiments (Fig. 1b; indicated by asterisks). We verified for one participant that the observed IFN-γ production was not a result of contamination with CD8 T cells by comparing two ELISpot assays, one with CD8 depleted PBMC and one with isolated CD8 T cells. The ELISpot assay with the isolated CD8 T cells showed IFN-γ production in response to different peptides compared to the assay with CD8 depleted PBMC, suggesting that the different epitopes were recognized in the context of MHC class-I and MHC class-II (Fig. 1c). Overall, these data indicated that with the possible exception of the peptides derived from *Candida* MP65, the peptide pools activated IFN-γ production in CD4 T cells, presumably in an antigen-specific manner.

### Virion-release from latently infected cells after antigenic stimulation measured by real-time RT-qPCR

For nine participants, we stimulated five million CD8 depleted PBMC with each peptide pool in quadruplicate for seven days in the presence of the integrase inhibitor raltegravir [1 µM]. Raltegravir prevented viral spread and ensured that the measured cell-free, virion-associated (cf)-RNA was released directly from reactivated reservoir cells and not a consequence of propagation of virus in the culture. Depending on the yield of isolated cells from each participant, we stimulated the cells with overlapping peptide pools from Gag, CMV, Candida, CEFT, Pol, Env, and Nef at a concentration of 1 µg/ml per peptide. DMSO was used as negative control and platebound antibodies against CD3 and CD28 were used as a maximum-stimulation control.

**Fig. 2:**
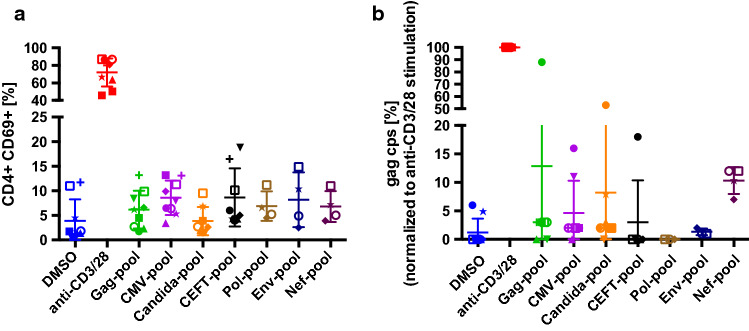
**Immune activation and latency reversal after stimulation of CD8 depleted PBMC with antigenic peptide pools measured by real-time RT-qPCR of released virions.** **a** Flow cytometric detection of CD69 expression levels on CD4 T cells after 48 hours of stimulation with the indicated peptide pools. All peptide pools were used at a concentration of 1 µg/ml per peptide. **b** Expression levels of cell-free HIV-1 Gag RNA after seven days of stimulation with the indicated peptide pools measured by real-time RT-qPCR detecting HIV-1 Gag (n = 9). Cells were incubated in the presence of raltegravir to prevent viral propagation. The values graphed are the means of quadruplicate samples (5 × 10^6^ CD8 depleted cells for each sample) for each participant and each condition. Each participant’s cells are shown using a distinct symbol; the open and closed circles are cells from the same participant obtained on two occasions approximately one year apart. Values were normalized to the positive control (antibodies to CD3 and CD69) for each participant. DMSO is the negative control

We first assessed the activity of the peptide pools with respect to immune activation by measuring the early activation marker CD69. After two days of culture, an aliquot of ~ 50,000 cells from each condition was stained for cell-surface CD69 and CD4 and analyzed by flow cytometry. The DMSO controls showed baseline CD69 positive CD4 T cells ranging from 1–12%, whereas the platebound anti-CD3 and anti-CD28-stimulated cells showed the maximum T cell activation for each participant, which ranged from 45–87% CD69 positive CD4 T cells (Fig. 2a). In general, none of the peptide pools activated more than 20% of the CD4 T cells.

After seven days of antigen-stimulation in the presence of raltegravir, we measured released virion-associated RNA to assess reversal of latency. The culture supernatants were cleared of debris and cellular contaminants by low-speed centrifugation, and the virions were isolated by ultracentrifugation through 20% sucrose. Cell-free (cf)RNA was isolated and cf-HIV-1 Gag RNA was measured using real-time RT-qPCR (Fig. 2b). The results were normalized to the cf-RNA yields after maximum stimulation with antibodies to CD3 and CD28 for each participant. One participant was evaluated twice, at times approximately one year apart (Fig. 2b, filled circles and open circles). At the first evaluation, this participant’s cells showed relatively high fractional levels of latency-reversal in response to several peptide pools including the Gag and CEFT pools, despite a limited up-regulation of CD69 (Fig. 2a, filled circles). At this time, this participant’s cells yielded cf-RNA in response to the *C. albicans* peptide pool, although this pool did not show activity in the IFN-γ ELISpot assay using cells from other participants (Fig. 1a). Also, at this time, this participant’s cells had the highest baseline expression of cf-RNA in the DMSO control. At the later time (open circles in Fig. 2b), this participant’s cells showed a different pattern of latency reversal: under 5% of the positive control values in all cases except for the Nef peptide pool, which was over 10% of the positive control value. Cells from the other participants shown in Fig. 2 yielded modest latency reversal after exposure to the peptide pools (less than 15% of the positive control values). The HIV-1 Nef peptide pool appeared the most consistent among multiple participants, although the fraction of latency reversal relative to the positive control was less than 15%.

### Viral cell-associated mRNA induction after antigenic stimulation measured by droplet digital (dd)PCR

**Fig. 3:**
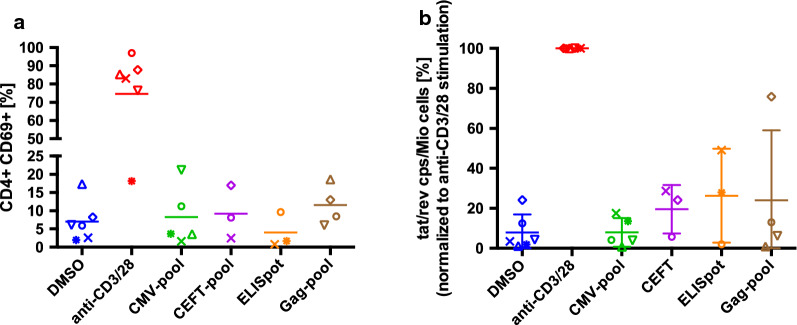
**Immune activation and latency reversal after stimulation of CD8 depleted PBMC with antigen peptide pools measured by droplet digital (dd)PCR of cellular Tat/Rev mRNA.** **a** Flow cytometric detection of CD69 expression levels on CD4 T cells after 48 hours stimulation with the indicated peptide pools. All peptide pools were used at a concentration of 1 µg/ml per peptide. **b** Expression levels of cell-associated multiply spliced HIV-1 Tat/Rev after five days stimulation with the indicated peptide pools (n = 6). Nine-million cells from each participant were stimulated as indicated, then plated in three-fold limiting dilutions and six replicates. “ELISpot” refers to a custom, participant-specific Gag-peptide pool selected by IFN-γ ELISpot assay of individual peptides. Cells were incubated in the presence of raltegravir to prevent viral propagation. Each participant’s cells are shown using a distinct symbol. Some participant’s cells were not tested with every peptide pool. Values were normalized to the positive control (antibodies to CD3 and CD69) for each participant. DMSO is the negative control

To confirm and extend the results obtained measuring cf-RNA, we evaluated latency reversal in a second group of participants by measuring cell-associated (ca) HIV-1 mRNA using a ddPCR assay that detects multiply spliced Tat/Rev transcripts [27]. We stimulated nine million CD8 depleted PBMC from six participants with each peptide pool, again in the presence of 1 µM raltegravir to prevent viral spread. Depending on the yields of participant cells, CD8 depleted PBMC were stimulated with peptide pools from CMV, CEFT, Gag, or a participant-customized ELISpot-selected Gag peptide pool at a concentration of 1 µg/ml and plated in three-fold limiting dilutions and six replicates. As before, we used DMSO as the negative control and platebound antibodies to CD3 and CD28 as a maximum-stimulation control. Cellular activation was measured by the up-regulation of CD69 after 48 hours of incubation. Consistent with the results above (Fig. 2a), we observed that the peptide pools did not activate more than 20% of the CD8 depleted PBMC (Fig. 3a).

After five days of culture in the presence of 1 µM raltegravir, we collected the cells and isolated ca-RNA to measure the amounts of multiply spliced Tat/Rev mRNA using the ddPCR assay. Overall, the ddPCR mRNA data showed no consistent induction of the expression of multiply spliced Tat/Rev mRNA by the different peptide pools. With some exceptions the peptide pools reversed latency only modestly when compared to the positive control (Fig. 3b). In cells from one participant (indicated by open diamond), the Gag peptide pool induced Tat/Rev mRNA to 75% of the positive control value, but for this participant the DMSO control value was also unusually high (24%). Notably, in cells from another participant (indicated by “x”), the ELISpot-selected Gag-peptide pool induced Tat/Rev mRNA to 50% of the positive control value, while the DMSO control value in this case was low (under 3%). This participant also had partial latency reversal in response to peptide pools of CMV (18% of the positive control) and the CEFT mixture (29% of the positive control).

### Antigen presentation by autologous monocyte-derived dendritic cells (DC)

**Fig. 4:**
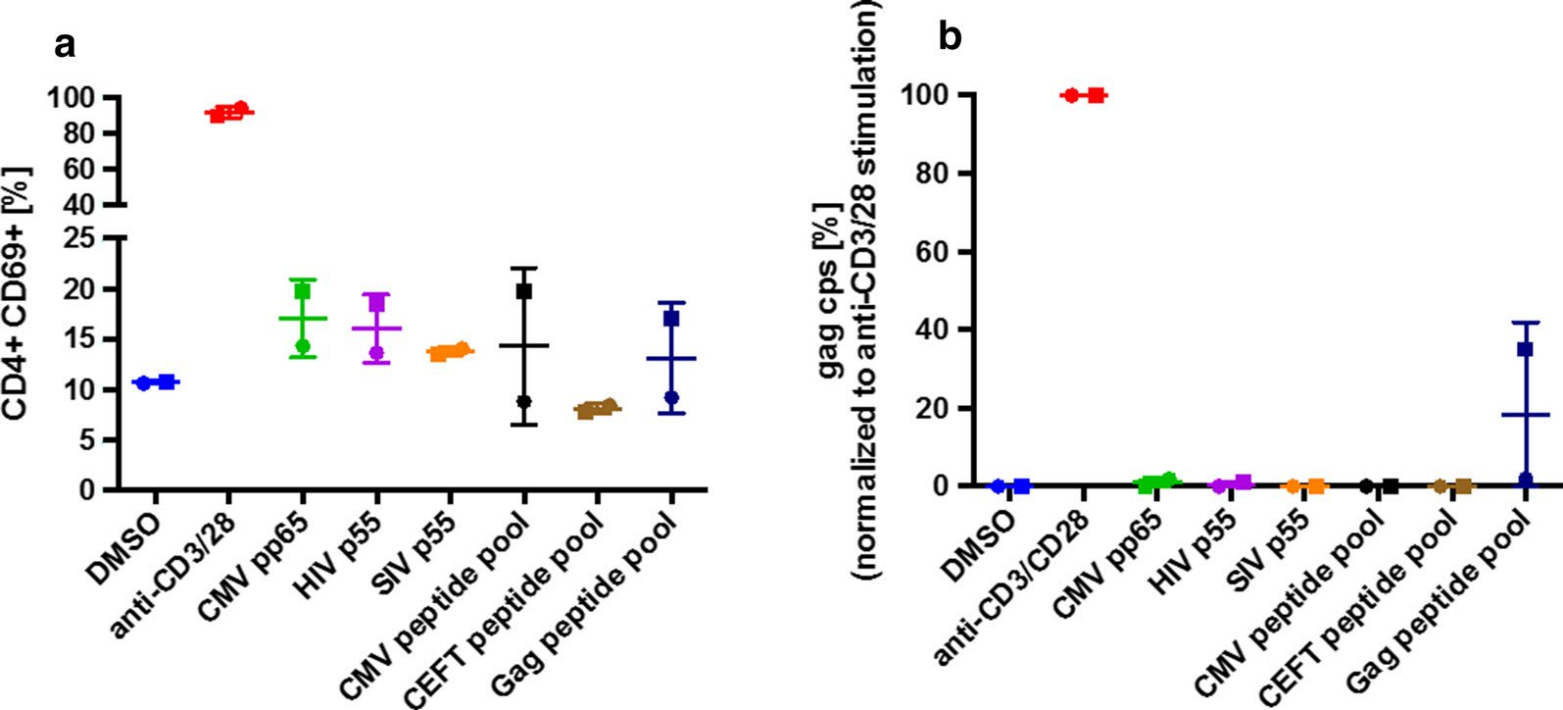
Latency reversal by co-culture with antigen-loaded dendritic cells (DC). ** a **Flow cytometric detection of CD69 expression levels on CD4 T cells after 48 hours stimulation with the indicated peptide pools or whole proteins and DCs. All peptide pools were used at a concentration of 1 µg/ml per peptide. **b** Expression levels of cell-free HIV-1 Gag RNA measured by real-time RT-qPCR after seven days of co-stimulation with DC presenting the indicated proteins and peptide pools at a concentration of 1 µg/ml per peptide in the presence of 1 µM raltegravir (RAL). Each participant’s cells are shown using a distinct symbol (n = 2)

We considered that suboptimal antigen presentation might render the above experiments less sensitive to the potential activity of antigens as LRAs. Therefore, we isolated, differentiated, and matured autologous monocyte-derived DC from two additional participants and used these mature DC as antigen-presenting cells. We also used complete proteins in addition to peptide pools as antigens to better simulate natural antigen processing and presentation (Fig. 4; Table 2). In these co-culture experiments, like those above, we included the integrase inhibitor raltegravir to block the spread of infection during the seven day incubation (Fig. 4b), but for one participant we also omitted the raltegravir and incubated the cells for 18 days to determine how allowing viral propagation would affect the results (Table 2).

**Table 2:** RNA measurements of latency reversal after presentation of the indicated antigens by dendritic cells for one of the participants shown in Fig. 4; cells were cultured with or without raltegravir

Assay	DMSO	α-CD3 + α-CD28	CMV pp65	HIV-1 p55 Gag	SIV p55 Gag	CMV pp65 peptide pool^a^	CEFT^b^ peptide pool	HIV-1 Gag peptide pool
cf^c^-RNA raltegravir^d^	n.d.^e^ (0)	24,000 (100)	n.d. (0)	130 (0.5)	n.d. (0)	107 (0.4)	n.d. (0)	8300 (35)
cf-RNA no raltegravir^f^	n.d. (0)	1.2 × 10^10^ (100)	n.d. (0)	1 × 10^10^ (87)	370 (3 × 10^− 6^)	440 (4 × 10^− 6^)	n.d. (0)	33,000 (2.7 × 10^− 4^)
ca^g^-RNA raltegravir^d^	n.d. (0)	151 (100)	3 (2)	n.d. (0)	n.d. (0)	14 (9)	30 (20)	96 (64)
ca^g^-RNA no raltegravir^f^	n.d. (0)	1.2 × 10^9^ (100)	n.d. (0)	7.5 × 10^6^ (0.63)	n.d. (0)	n.d. (0)	n.d. (0)	50 (4 × 10^− 6^)

Values in parentheses are the percent of the α-CD3/α-CD28 control

^a^All peptide pools were used at 1 µg/ml each peptide

^b^CEFT, pool of MHC class-II restricted peptides from CMV, EBV, influenza virus, and tetanus toxoid

^c^cf, cell-free, virion-associated RNA in copies/ml

^d^Raltegravir was used at 1 µM to block viral propagation and the cells were stimulated for 7 days

^e^n.d., not detected

^f^Cells were stimulated for 18 days in the absence of raltegravir

^g^ca, cell-associated, Tat/Rev mRNA in copies/µg RNA

Consistent with our experiments using CD8 depleted PBMC, the use of autologous DCs to present antigen did not cause substantial T cell activation measured by CD69 up-regulation (Fig. 4a), nor did it yield consistent latency reversal by any peptide or protein antigen (Fig. 4b). For both participants, we used cf-RNA for the initial readout (Fig. 4b). The data of one participant indicated that in the presence of raltegravir, the Gag peptide pool reversed latency to almost 40% of the positive control value, but the other peptide pools and complete proteins were inactive.

For the participant in whom the Gag peptide pool appeared active (square symbol in Fig. 4b), we also omitted raltegravir from parallel cultures: in this condition the intact p55 Gag protein yielded substantial cf-RNA (1 × 10^10^ copies/ml) reflecting marked viral outgrowth, but neither SIV Gag nor any other antigen did (Table 2). Notably, although the Gag peptide pool did not yield amounts of cf-RNA comparable to maximum-stimulation or the p55 Gag protein, it differed from the other conditions, yielding 3 × 10^4^ copies/ml of cf-RNA compared to the low or undetectable copies for DMSO and the other antigens (Table 2). For this participant (square symbol in Fig. 4b), we also used ddPCR of ca Tat/Rev mRNA as the readout (Table 2). The data indicated that in the presence of raltegravir, the CMV, CEFT, and Gag peptide pools each reversed latency substantially. The activity of the Gag peptide pool (64% of maximum-stimulation) measured by the ca-RNA (Tat/Rev mRNA) readout in the presence of raltegravir was consistent with that measured using the cf-RNA (virion-RNA) readout (35% of maximum-stimulation). In contrast, when raltegravir was omitted, only HIV1 p55 protein yielded detectable induction of Tat/Rev mRNA: stimulation with p55 yielded 7.5 × 10^6^ copies/ml compared to 1.2 × 10^9^ copies/ml after maximum stimulation. Overall, these results suggested that antigen presentation by DCs did not markedly increase the degree or consistency of latency reversal by these peptides and proteins. The results also suggested that prolonged culture can change the conclusions of these latency reversal experiments relative to short-term, “single-cycle” readout, either amplifying or reducing the signals.

## Discussion

We sought to determine whether the viral reservoir resides in latently infected CD4 T cells of a limited specificity and whether the reservoir can be reactivated with specific antigens. We used two different methods of antigenic stimulation and two different assays to detect latency-reversal. While previous studies have suggested the potential of HIV-1 peptides as LRAs using ex vivo CD4 positive T cells from participants with prolonged suppressed viremia [22, 23], our results indicate that the inducible viral reservoir does not consistently reside in HIV-specific cells. Nonetheless, in some participants herein (three of sixteen), Gag and Gag-peptides were relatively effective at reversing latency.

The IFN-γ ELISpot assay confirmed that the antigenic peptide pools used in these experiments induced IFN-γ production in CD8-T cell-depleted PBMC, but in general they did not consistently induce substantial latency-reversal. In the case of Gag peptides, latency-reversal was not substantially improved by “customizing” the peptide pool by including only the peptides to which the participant had an ELISpot response, although only three participants were tested with such customized pools. Apparently, the antigens tested induced at best only partial reactivation of the latent reservoir. One study demonstrated that even maximum CD4 T cell activation did not induce the entire replication-competent latent reservoir and that repeated stimulation increased the extent of latency-reversal [28]. In this regard, our experimental design provided the possibility for multiple cell activation events, since the CD8 depleted PBMC were cultured for five to seven days with the antigens. Alternatively, the latent reservoir might be differentially distributed among memory CD4 T cells with distinct specificities in different participants, and it might include naïve CD4 T cells. In these scenarios, results such as those obtained here would be expected.

In case the method of antigen-presentation used for most of our study - using peripheral blood monocytes and B cells loaded with peptides—was suboptimal, we tested co-culture of CD4 T cells with differentiated and matured monocyte-derived autologous DC that were loaded with antigenic peptide pools or proteins. DC are professional antigen presenting cells and efficiently induce CD4 and CD8 T cell responses [29]. A major function of DC is to interact with CD4 T cells and present antigen, and DC have been linked to both cell-mediated HIV-1 infection as well as the establishment of latency [30–32]. Moreover, a recent study supports that autologous monocyte derived DC can enhance antigen-presentation to reverse latency [24]. Nonetheless, a potential confounder in the design of that study was that the cultures were maintained in the absence of antiretroviral drugs and the viruses reactivated from latency were allowed to spread ([29] and reviewed in [25]). This allows the readout to reflect not only the initial reversal of latency, but also the ability of the culture to amplify the virus, an ability that likely is in turn due to the extent of cellular activation. For example, if a large number of cells respond to a given antigen, but only a small fraction of them harbor latently infected cells, then a substantial signal for “latency reversal” can nonetheless result from amplification due to viral replication. We potentially observed this effect in one of our participants whose DCs were loaded with p55 protein, which yielded a very low signal in the presence of raltegravir but a robust signal in its absence. We also observed the opposite effect in the same participant in the case of the CEFT peptide pool: low signal in the cell-associated Tat/Rev mRNA assay in the presence of raltegravir, but none in its absence. In principle, this latter discordance could be due to transient production of mRNA from viral genomes that are defective. Alternatively, both of these types of discordant results could be due to stochastic distributions of cells of different antigen specificity among the samples that are intended as replicates, but due to the very low fraction of latently infected cells might in fact yield variable results.

Our study has several caveats. The number of participants is small, especially regarding the use of dendritic cells, where only two participants were tested. Although we confirmed that most of the peptide pools stimulated at least some of the cells under the conditions tested, these conditions or the pools themselves might yet be suboptimal for antigen-presentation. Despite the use of 5–9 million CD8 depleted PBMC per condition tested, stochastic variation among the wells treated with different antigens could affect the results as noted above. Notably, in two of the three examples of substantial latency reversal by Gag-peptides, the DMSO control values were unusually high; this suggests an unknown condition that might have biased those results, such as medical non-compliance before the sample was obtained. In one of those cases, a repeat analysis of the participant one year later yielded lower values after stimulation with the DMSO control and the Gag-peptides, consistent with this possibility.

Taken together our results suggest a high complexity of the latent viral reservoir with respect to its distribution among antigen-specific CD4 T cells. The reservoir does not seem concentrated in a CD4 T cell subset specific for a certain antigen, but rather is likely located in CD4 T cells of diverse specificity. A minority of instances herein supported preferential residence of the reservoir in HIV-specific cells, but the majority did not. The occasional participants in whom the reservoir does seem concentrated in specific cells are consistent with the notion of clonal expansion of latently infected cells in response to specific antigen. Alternatively, the reservoir in these participants could be coincidentally concentrated in a subset of CD4 T cells that are specific for a certain antigen.

Importantly, the timing of ART-initiation and in particular the duration of chronic infection before cART might influence the antigen-specificity of the reservoir-cells. For example, in participants who initiate cART promptly during primary infection, the fraction of reservoir cells specific to HIV-1 might be the highest. This hypothesis remains important to test, because it could provide a path to latency-reversal with HIV-1 antigens and potential eradication in that specific subset of patients.

## Conclusions

Stimulation of T cell responses by peptide or protein antigens reversed HIV-1 latency ex vivo, but rarely to a substantial extent, and no particular antigen was active in all cases. When the reversal of latency by peptides or proteins occurred, the extent was greater than would be predicted based on the extent of T cell-activation. In these individuals, the latent reservoir seems disproportionally present in cells of specific antigen-reactivity, a finding potentially consistent with clonal expansion of specific memory CD4 T cells.

## Methods

### Study participant samples

We received up to 300 ml blood whole from 19 study participants with the following inclusion criteria: HIV-1 positive, suppressed viremia (< 50 cps/ml blood) for at least one year, and a CD4 T cell count of at least 500 cells/µl blood. Clinical data is shown in Table 1.

### Isolation of PBMC, CD8 T cell depletion, and stimulation with peptide pools

Peripheral blood mononuclear cells (PBMCs) were isolated from up to 300 ml whole blood donations using Lymphoprep (Stemcell Technologies) and density gradient centrifugation. CD8 T cells were depleted by positive selection using the EasySep™ Human CD8 Positive Selection Kit (Stemcell Technologies) and EasySep™ magnet (Stemcell Technologies). Depletion of CD8 T cells was confirmed by staining an aliquot of 50,000 CD8 depleted PBMC with anti-CD3-FITC (clone: OKT3), anti-CD4-PE (clone: OKT4) and anti-CD8-APC (clone: HIT8a) fluorescent antibodies and analysis with an Accuri C6 flow cytometer and software (BD Biosciences).

Antigen reactivity of CD8 T cell depleted PBMC to the peptide pools was determined where indicated by IFN-γ ELISpot assay (see below), and cells were subsequently stimulated with the various antigen peptide pools (see below) at 1 µg/ml each peptide. After 48 hours of antigen stimulation, an aliquot of ~ 50,000 CD8 depleted PBMC of each condition was stained for anti-CD4-APC (clone: OKT4) and anti-CD69-PE (clone: FN50) and analyzed using the Accuri C6 flow cytometer and software (BD Biosciences). All antibodies for flow cytometry were obtained from BioLegend.

### Peptides and peptide pools

For antigen stimulation of CD8 depleted PBMC we used pools of 15mer peptides overlapping by 11 amino acids. These peptide pools were specific for: HCMV pp65 (138 peptides), PepMix Candida (MP65) (JPT—92 peptides), PepMix CEFT-MHC-II-pool (JPT—4 peptides selected from defined HLA class II-restricted T cell epitopes of *Clostridium tetani*, Epstein-Barr virus (HHV-4), Human cytomegalovirus (HHV-5), and Influenza A), HIV-1 consensus B Gag (123 peptides), HIV-1 consensus B Env (211 peptides), HIV-1 consensus B Pol (249 peptides), and HIV-1 consensus B Nef (49 peptides). Peptides were > 80% pure. Stock solutions were made at a concentration of 5 mg/ml reconstituted in DMSO for all peptides and peptide pools, and further diluted with R10 media to generate working stocks at a concentration of 200 µg/ml. The 123 peptides of HIV-1 consensus B Gag peptide-pool were also tested individually using an IFN-γ ELIspot assay (see below).

For experiments using matured monocyte-derived DC, we used proteins of CMV pp65 (Miltenyi Biotec), HIV-1 Gag p55 (Abcam) and SIV Gag p55 (Proteinsciences) as a control.

The following reagents were obtained through the NIH AIDS Reagent Program, Division of AIDS, NIAID, NIH: HCMV pp65 Peptide Pool (cat# 11549) [33–35], HIV-1 Consensus B Gag Peptide Pool (cat# 12425), HIV-1 Consensus B Gag Peptide Set (cat# 8117), HIV-1 Consensus B Pol Peptide Pool (cat# 12438), HIV-1 Consensus Subtype B Env Peptide Pool (cat# 12540), HIV-1 Consensus Subtype B Nef Peptide Pool (cat# 12545), and HIV-1 Consensus Subtype B Nef Peptide Set (cat# 5189).

### Generation of mature monocyte derived dendritic cells (DC)

CD14 monocytes were isolated from PBMC using magnetically labelled anti-CD14 antibodies (Stemcell Technologies). Monocytes were cultured for 5 days in Iscove’s Modified Dulbecco’s Media (Gibco) complemented with 10% fetal bovine serum, penicillin/streptomycin (Gibco), granulocyte-monocyte colony-stimulating factor (GM-CSF, 1000 IU/ml, R&D Systems), and interleukin4 (IL-4, 1000 IU/ml, R&D Systems). On day five, maturation factors [interferon (IFN)-α (1000 IU/ml, R&D Systems), IFN-γ (1000 U/ml, R&D), IL-1β (10 ng/ml, R&D Systems), tumor necrosis factor (TNF)-α (25 ng/ml, R&D Systems), and polyinosinic:polycytidylic acid (20 ng/ml, Sigma-Aldrich)] were added to the cultures for 48 hours, as previously described [36]. DC-maturation was confirmed by staining the cells with antibodies against CD11c, CD80, CD83, CD86, CD143, CD169, CD197 (all antibodies were obtained from Biolegend) and analysis by flow cytometry. Mature DC were loaded with the antigen for two hours, washed in IMDM media and subsequently co-cultured with autologous CD4 T cells in complete IMDM in the presence of the integrase inhibitor raltegravir [1 µM] at a ratio of 1:10 (100,000:1 million) for seven days (or in the absence of raltegrvir for 18 days) in 12-well plates. Culture supernatants and cells were collected on day seven (presence of raltegravir) or day 18 (absence of raltegravir) for quantitation of HIV-1 cf-RNA and ca-RNA.

### Interferon-gamma ELISpot assay

Freshly isolated CD8 depleted PBMC were seeded in 96-well polyvinylidene difluoride-backed plates (MAIP S45, Millipore) that had been coated with an anti-IFN-γ MAb 1-D1k (0.5 µg/ml, Mabtech) overnight at 4 °C.

HIV-specific CD4 T-cell responses were quantified, as described previously [37]. Overlapping peptides were added to 1 × 10^5^ CD8 depleted PBMC per well at a final concentration of 12.5 µg/ml. The plates were then incubated for a 14–16 hours at 37°C and 5% CO_2_. IFN-γ positive were detected using a biotinylated secondary anti-IFN-γ MAb 7-B6-1 (0.5 µg/ml, Mabtech), a streptavidin-alkaline phosphatase conjugate (Mabtech) and TMB substrate (Mabtech).

IFN-γ-producing cells were counted and expressed as spot-forming cells (SFC) per 10^6^ PBMC. Negative controls were always < 10 SFC per 10^6^ cells. As positive controls, we incubated PBMC with phytohemagglutinin or anti-CD3/CD28 coated beads. Wells were considered positive if they had at least 20 SFC/10^6^ PBMC.

### Real-time qRT-PCR

Five million CD8 depleted PBMC were stimulated with the respective peptide pools at a concentration of 1 µg/ml and plated on a 6-well cell culture plate. After seven days of culture in the presence of the integrase inhibitor raltegravir [1 µM] supernatant was collected from each well and centrifuged for 5 min at 300 x *g* to remove cells and debris. Virions were harvested by centrifugation of cell-free culture supernatants through a 20% sucrose cushion at 23,500 x *g* for one hour at 4 °C. The pelleted virus particles were resuspended in 200 µl PBS and cell-free (cf)-RNA was isolated using the Viral RNA extraction Kit (Roche) following the manufacturer’s instructions. Cell free viral RNA was eluted in 20 µl and real-time RT-qPCR was performed using Superscript III Platinum One-step qRT-PCR Kit (Invitrogen) and 10 µl cf-RNA, in duplicate. The PCR reactions used Cy5-labeled *gag* probes together with the corresponding forward and reverse primers. Cycling was performed in an 7900HT Sequence Detection System (Applied Biosystems) with the following parameters: 50 °C for 15 min for the RT-step and 95 °C for 2 min, followed by 50 cycles of 95 °C for 15 s and 60 °C for 30 s, and a final step of 50 °C for 10 min. A standard curve of pNL4-3 plasmid DNA in duplicate, 10-fold serial dilutions was included on each plate, as well as water negative controls. The limit of detection of this assay was 100 copies/ml *gag* DNA.

The following reagent was obtained through the NIH AIDS Reagent Program, Division of AIDS, NIAID, NIH: Raltegravir from Merck & Company, Inc (Cat # 11680).

### Droplet digital PCR

Nine million CD8 depleted PBMC were stimulated in the presence of 1 µM raltegravir with the respective peptide pools at a concentration of 1 µg/ml and plated on a 96-well plate with seven serial three-fold dilutions, starting with a concentration of 1 × 10^6^ CD8 depleted PBMC per well and six replicates for each dilution. After seven days the cells were pelleted, and ca-RNA was extracted using MagMAX-96 Total RNA Isolation Kit (Thermo Fisher) and the KingFisher Flex Magnetic Particle Processor (Thermo Fisher) following manufacturer’s instructions. Isolated HIV-1 RNA was reverse transcribed using the iScript Advanced cDNA Synthesis Kit (BioRad) and the following conditions: 46 °C for 20 min, followed by 95 °C for 1 min. The generated cDNA was subsequently subjected to ddPCR. The sample cDNA and ddPCR reaction mixture was loaded into a QX-200 emulsification device (Bio-Rad) and droplets were formed following the manufacturer’s instructions. Samples were transferred into a 96-well reaction plate and sealed with a pre-heated 96-well heat sealer (Eppendorf) for 2 s. Each reaction consisted of a 20 µl solution containing 10 µl ddPCR Probe Supermix, 900 nM primers, 250 nM probe, and template DNA with the following cycling conditions: 10 min at 95 °C, 40 cycles each consisting of a 30 s denaturation at 94 °C followed by a 58 °C extension for 60 s, and a final 10 min at 98 °C. After cycling, droplets were analyzed immediately or stored at 4 °C overnight until analysis. An aliquot of the isolated DNA was used for host cell RPP30 (Ribonuclease subunit P30) ddPCR and cycled with same parameters described above. Copy numbers were calculated as the mean of the three ddPCR replicate measurements and normalized to one million CD8 depleted PBMC as determined by RPP30 levels. The limit of detection of the ddPCR assay for HIV-1 DNA using the same primer-probe set was previously described as 0.7 copies per million of cells [38]. The detected number of RPP30 copies in each ddPCR reaction was used to estimate the number of cells per aliquot.

## Data Availability

Source data available on request.

## Abbreviations

ddPCR: Droplet digital PCR
DMSO: Dimethylsulfoxide
LRA: Latency reversal agent
PBMC: Peripheral blood mononuclear cells
